# A Novel Multiplex Real-Time PCR Assay for the Concurrent Detection of Hepatitis A, B and C Viruses in Patients with Acute Hepatitis

**DOI:** 10.1371/journal.pone.0049106

**Published:** 2012-11-08

**Authors:** Yongjung Park, Beom Seok Kim, Kyu Hun Choi, Dong Ho Shin, Mi Jung Lee, Yonggeun Cho, Hyon-Suk Kim

**Affiliations:** 1 Department of Laboratory Medicine, Severance Hospital, Yonsei University College of Medicine, Seoul, Republic of Korea; 2 Department of Internal Medicine, Severance Hospital, Yonsei University College of Medicine, Seoul, Republic of Korea; University of Cincinnati College of Medicine, United States of America

## Abstract

A novel multiplex real-time PCR assay for concurrent detection of hepatitis viruses was evaluated for its clinical performance in screening patients with acute hepatitis. A total of 648 serum samples were collected from patients with acute symptoms of hepatitis. Concurrent detection of nucleic acids of HAV, HBV and HCV was performed using the Magicplex™ HepaTrio Real-time Detection test. Serum nucleic acid levels of HBV and HCV were also quantified by the Cobas® AmpliPrep/Cobas® TaqMan® (CAP/CTM) HBV and HCV tests. Patients’ medical records were also reviewed. Concordance rates between the results from the HepaTrio and the CAP/CTM tests for the detection of HBV and HCV were 94.9% (*k* = 0.88) and 99.2% (*k* = 0.98), respectively. The cycle threshold values with the HepaTrio test were also correlated well with the levels of HBV DNA (*r* = −0.9230) and HCV RNA (*r* = −0.8458). The sensitivity and specificity of the HepaTrio test were 93.8% and 98.2%, respectively, for detecting HBV infection, and 99.1% and 100.0%, respectively, for HCV infection. For the HepaTrio test, 21 (3.2%) cases were positive for both HBV and HCV. Among the positive cases, 6 (0.9%) were true coinfections. This test also detected 18 (2.8%) HAV positives. The HepaTrio test demonstrated good clinical performance and produced results that agreed well with those of the CAP/CTM assays, especially for the detection of HCV. This assay was also able to detect HAV RNA from anti-HAV IgM-positive individuals. Therefore, this new multiplex PCR assay could be useful for the concurrent detection of the three hepatitis viruses.

## Introduction

Hepatitis A, B, and C viruses (HAV, HBV, and HCV) are common pathogens that cause hepatitis in humans. Approximately two billion people worldwide have been infected with HBV and about 350 million suffer from chronic hepatitis B (CHB). Moreover, about 25% of adults who were infected with HBV during childhood die from hepatocellular carcinoma (HCC) or liver cirrhosis [1]. In addition, an estimated three to four million people worldwide are infected with HCV each year, and 130 to 170 million people are chronically infected with HCV. More than 350,000 people die from HCV-related liver diseases each year [2]. HAV infections, which occur sporadically and in epidemics, account for an estimated 1.4 million worldwide cases annually [3].

In the United States, the prevalence of anti-HCV antibodies (anti-HCV) was about 1.6% between 1999 and 2002, which was an estimated 4.1 million people [4]. The prevalence of HCV infections was approximately 1% in Korea in 2003, but was 42.3% and 80.0% among hemophiliacs and intravenous drug users, respectively [5]. In a previous study, HBV and HCV infections, respectively, were associated with 68.1% and 15.2% of HCC cases in men and 69.9% and 18.8% in women [6]. Therefore, HBV and HCV infections are worrisome public health problems.

In recent years, antiviral therapies for CHB using nucleos(t)ide analogues and/or interferons have become standard treatments [7]. Many hepatologists have also treated chronic hepatitis C with interferon-alpha plus ribavirin, and there have been many efforts to develop new antiviral agents for the treatment of HCV infections [8]. Therefore, early and accurate diagnoses of HBV and HCV infections are essential for effective treatment and prevention of disease transmission.

HBV and HCV share common modes of transmission, and thus they can cause coinfection. In some previous studies, HCV coinfection rates among hepatitis B surface antigen (HBsAg)-positive individuals ranged from 3% to 18%, varying in the regions and subjects studied [9]–[11]. In another study, about 25% of hepatitis C patients were positive for HBV serologic markers, and this proportion was nearly six times higher than that of HCV-uninfected populations [12].

Although there have been some efforts to develop alternative assays to PCR tests for detecting hepatitis viruses [13]–[16], the diagnosis and monitoring of hepatitis B and C are usually based on PCR assays. In this context, a multiplex PCR assay for the concurrent detection of hepatitis viruses would be useful in screening for hepatitis virus infections as well as in detecting coinfection with different types of hepatitis viruses. Recently, the Korea Food and Drug Administration approved a multiplex real-time PCR test for use as an *in vitro* diagnostic test for the concurrent detection of HAV, HBV, and HCV. In this study, we evaluated the clinical performance of this novel multiplex PCR assay. We also assessed the clinical performance of this assay in screening for hepatitis from an independent high-risk population of blood-borne infections.

## Materials and Methods

### 1. Ethics Statement

The Institutional Review Board (IRB) of Severance hospital approved this study. Obtaining informed consents from the enrolled subjects was exempted by the IRB because this study was retrospectively performed by analyzing medical records and assaying stored residual specimens that had been requested for hepatitis virus PCR assays, and no additional specimen was collected from the subjects.

### 2. Samples

Between January 2009 and December 2011, a total of 648 serum samples, which were requested for the HBV or HCV PCR assay, were collected from patients who presented with current or recent past non-specific symptoms of hepatitis and/or who showed unexplained abnormal serum levels of liver enzymes but did not have a previous history of either hepatitis A, B or C at Severance Hospital, Yonsei University College of Medicine. All the samples were aliquoted and stored at −70°C within 1 hour after arrival. Concurrent detection of HAV, HBV, and HCV from all 648 samples was performed using the Magicplex™ HepaTrio Real-time Detection (HepaTrio) test (Seegene Inc., Seoul, Korea). The results of previous PCR tests for the respective samples were blinded during the entire assay process of the HepaTrio test.

### 3. Multiplex Real-time PCR Assay

The HepaTrio test is a commercial semi-automated nested multiplex real-time PCR assay, which uses four kinds of fluorophores for the concurrent detection of amplicons from HAV, HBV, HCV, and the Whole Process Control material (WPC). This assay utilizes a unique technology, Dual Priming Oligonucleotide (DPO™). The DPO is composed of a longer 5′-segment (stabilizer), a shorter 3′-segment (determiner), and a poly (I) linker that bridges these two segments. The linker generates a bubble-like structure which itself is not involved in priming, but separates out the two parts of the primer to assign the two primer segments to distinct annealing properties: stable priming and target-specific extension. This allows blocking of extension of non-specifically primed templates and improves the PCR specificity [17], [18]. The target of the primers for the amplification of HAV and HCV is 5′-untranslated regions (UTR), which are known to be highly conserved among the genotypes, especially genotypes 1 to 6 in the case of HCV, and the target region of HBV is *N*-terminal of *S* gene, which is also known as highly conserved along with genotypes A to H and unrelated with drug resistance mutations of HBV.

Nucleic acids were eluted from 500 µL of sample to 100 µL of solution using the MICROLAB® STARlet automated purification system (Hamilton Company USA, Reno, NV, USA). Reverse transcription PCR comprising 45 cycles was performed using the Magicplex™ HepaTrio Amplification kit (Seegene, Inc.) and the amplified products were analyzed by 20 cycles of real-time PCR using the Magicplex™ HepaTrio Real-time Detection kit (Seegene Inc.) and the CFX96™ Real-Time PCR Detection System (Bio-Rad Laboratories, Inc., Hercules, CA, USA). The fluorescene signal from PCR amplification was automatically interpreted to the result by using the CFX Manager™ software version 2.1 (Bio-Rad Laboratories, Inc.) based on the pre-assigned experiment protocol and cutoff cycle threshold values (Ct) suggested by the manufacturer as 10.0, 14.0, and 12.0, respectively, for HAV, HBV, and HCV. Samples showing Ct of equal or less than these values were regarded as positive for the nucleic acids of the respective viruses. To ensure that all assay processes were properly performed, the WPC provided with the assay kit was added to the samples before the nucleic acid extraction, and the results were regarded as valid when the Ct for the WPC was equal to or less than 5.0. The whole assay process was repeated when the result for WPC was invalid. Positive and negative control materials provided with the HepaTrio kits were also included in each test batch.

### 4. Viral Load Quantification

Levels of HBV DNA and HCV RNA in the same specimens were quantified using the Cobas® AmpliPrep/Cobas® TaqMan® (CAP/CTM) HBV Test v2.0 and the CAP/CTM HCV test (Roche Molecular Diagnostics, Pleasanton, CA, USA), respectively, which are fully automated real-time PCR assays. All assays were performed according to the manufacturer’s instructions. The lower and upper detection limits of the CAP/CTM HBV test are 20 IU/mL and 1.7×10^8^ IU/mL HBV DNA, respectively, and those of the CAP/CTM HCV test are 15 IU/mL and 6.9×10^7^ IU/mL HCV RNA, respectively. Results below the lower detection limits were considered negative.

### 5. Other Assays

HBsAg, antibodies to HBsAg (anti-HBs) and antibodies to HBV core antigen (anti-HBc) in a serum sample were detected using an ARCHITECT® i4000SR analyzer with respective assay reagents (Abbott Laboratories, Abbott Park, IL, USA). Anti-HCV was detected using the Cobas® e 411 automated immunoassay analyzer with the Elecsys® Anti-HCV reagent kit (Roche Diagnostics GmbH, Mannheim, Germany). The presence of IgM antibodies to HAV (anti-HAV IgM) in a sample was determined using the Vitros® 3600 Immunodiagnostic System with an anti-HAV IgM reagent kit (Ortho-Clinical Diagnostics, Johnson & Johnson Co., Buckinghamshire, UK). Serum levels (IU/L or mg/dL) of aspartate aminotransferase (AST), alanine aminotransferase (ALT), alkaline phosphatase (ALP) and total bilirubin were measured by the Hitachi 7600 DDP modular chemistry analyzer (Hitachi High-Technologies Co., Tokyo, Japan). Normal upper reference limits for AST and ALT in our laboratory were 34 and 46 IU/L, respectively. HCV genotypes were determined by the LINEAR ARRAY HCV Genotyping test (Roche Molecular Diagnostics).

### 6. Diagnostic Accuracy Criteria

The ‘diagnostic accuracy criteria’ [19], used to determine whether patients were infected with HBV or HCV, were based on following criteria. When the results of the HepaTrio and the CAP/CTM tests for HBV or HCV were concordant, they were regarded as true-positive or -negative. When there were discrepancies between the HepaTrio and CAP/CTM test results, the true diagnosis was determined by retrospective blind review of the patient’s medical records, including assessment of repetitive serologic and PCR test results between one month before and after sample collection. Patients were considered uninfected with HBV when they were repetitively negative for HBsAg and HBV DNA and either positive for anti-HBs or negative for anti-HBc along with normal serum aminotransferase levels. The patients were also considered uninfected with HCV when the anti-HCV assays as well as follow-up CAP/CTM HCV tests were all repetitively negative along with normal serum aminotransferase levels. When a patient could not be classified based on the serologic and PCR test results, the diagnosis was determined to be ‘indeterminate’ and was excluded from estimating clinical performance.

### 7. Validation of the Clinical Performance of the HepaTrio Test

To validate the clinical performance of the HepaTrio test for detecting HBV and HCV infections from a population with high risk of blood-borne infections, a total of 100 serum samples were collected from sequential patients who visited Severance Hospital to receive regular hemodialysis between January and February 2012. All the samples were assayed using the HepaTrio test. Retrospective blind reviews of the medical records for all those patients were performed. A patient was regarded as having true HBV infection when a sample drawn between one month prior and after the sample collection for this study was positive for HBV DNA by the CAP/CTM HBV test, at the same time, HBsAg and anti-HBs were respectively positive and negative two or more times. Patients were considered to be not infected with HBV either when two or more of the HBsAg and anti-HBs assays were negative and positive, respectively, concurrent with normal serum aminotransferase levels, or when two or more of the HBsAg and anti-HBc assay results were all negative and serum aminotransferase levels were normal during the most recent three months.

A patient was defined as having true HCV infection if HCV RNA was detected by the CAP/CTM HCV test in a sample collected during one month before and after the specimen used in this study was drawn, concurrent with two or more positive results on anti-HCV assays. The patients were regarded as uninfected with HCV when two or more results of the anti-HCV assay were negative during the most recent three months, with normal serum aminotransferase levels.

### 8. Statistical Analysis

Analyse-it Method Evaluation Edition version 2.27 software (Analyse-it Software Ltd., City West Business Park, Leeds, UK) was used for all statistical analyses. The concordance rate and kappa coefficient (*k*) with its 95% confidence interval (CI) were calculated to estimate the concordance between the results of different assays. Correlation coefficients (*r*) between the Ct obtained by the HepaTrio test and viral loads quantified by the CAP/CTM assays were assessed by the Spearman’s rank test. Sensitivities, specificities and their respective 95% CIs were calculated based on the ‘diagnostic accuracy criteria’ described above. Pairwise comparison of HCV RNA levels between the HCV genotype groups were performed using Kruskal-Wallis test with Bonferroni correction to compensate alpha statistical error from multiple comparisons. A *P*-value of less than 0.05 was considered significant.

## Results

### 1. Subject Characteristics

The total of 648 patients suspected of having acute hepatitis included 340 males (52.5%) and 308 females (47.5%). The median age of the subjects was 54.1 years (first to third quartiles, 42.1 to 65.0 years). Median serum levels of ALT, AST, ALP and total bilirubin at the patient’s first visit were 112.0 IU/L (first to third quartiles, 66.0 to 227.3 IU/L; maximum 7,827 IU/L), 94.0 IU/L (first to third quartiles, 56.7 to 192.0 IU/L; maximum 9,423 IU/L), 70.0 IU/L (first to third quartiles, 56.9 to 94.0 IU/L; maximum 427 IU/L), and 0.9 mg/dL (first to third quartiles, 0.6 to 1.3 mg/dL; maximum 28.1 mg/dL), respectively.

**Figure 1:**
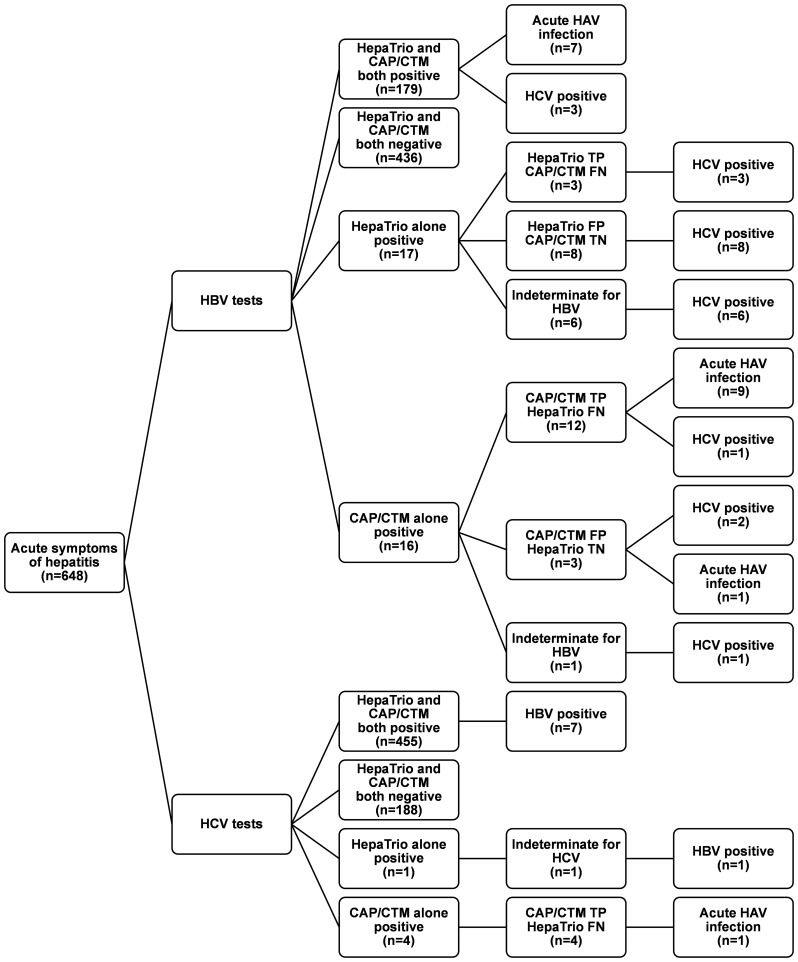
Results of the HepaTrio and the Cobas® AmpliPrep/Cobas® TaqMan® (CAP/CTM) HBV and HCV tests. For discrepant cases between the HepaTrio and the CAP/CTM tests, medical records including the results of serologic tests were reviewed. TP, true positive; FN, false negative; FP, false positive; TN, true negative.

Infections with one or more types of hepatitis virus were identified in all subjects (Figure 1). The median HBV DNA concentration in the 194 cases of true HBV infection was 2.35×10^4^ IU/mL (first to third quartiles, 6.13×10^2^ to 1.75×10^7^ IU/mL), and the median Ct in the HepaTrio test was 4.89 (first to third quartiles, of 3.66 to 7.27). In addition, the median level of HCV RNA in the 459 cases of true HCV infection was 1.51×10^6^ IU/mL (first to third quartiles, 2.04×10^5^ to 5.32×10^6^ IU/mL), and the median Ct in the HepaTrio test for these patients was 4.15 (first to third quartiles, 3.56 to 4.88). Among the HCV infection cases, 311 samples were genotyped for HCV, and 163 (52.4%), 133 (42.8%), and 15 (4.8%) of them were genotype 1, 2, and 3, respectively. Median HCV RNA level of cases infected with HCV genotype 1 was 2.45×10^6^ IU/mL (first to third quartiles, 6.54×10^5^ to 6.32×10^6^ IU/mL) and was higher than those with genotype 2 (median, 9.78×10^5^ IU/mL; first to third quartiles, 1.25×10^5^ to 3.73×10^6^ IU/mL; *P* = 0.0006) and genotype 3 (median, 1.24×10^5^ IU/mL; first to third quartiles, 4.88×10^4^ to 2.85×10^5^ IU/mL; *P*<0.0001). HCV RNA levels were also higher in genotype 2 infections than in genotype 3 infections (*P* = 0.0195).

### 2. Concordance between the Test Results

Agreement between the results from the HepaTrio and the CAP/CTM tests is presented in Table 1. The results from the HepaTrio test for detecting HBV DNA or HCV RNA agreed with those from the CAP/CTM HBV or HCV test in 94.9% and 99.2% of all 648 cases, respectively. Detailed results for the discrepant cases between the CAP/CTM and the HepaTrio tests are summarized in Table 2.

**Table 1:** Agreement between the results of the HepaTrio test and the CAP/CTM HBV and HCV assays.

		HepaTrio						
Target virus	CAP/CTM	Negative	Positive	Total	Concordance rate (%)	Kappa coefficient	95% CI	*P*-value
HBV	Negative	436	17	453				
	Positive	16	179	195				
	Total	452	196	648	94.91	0.8791	0.8390 to 0.9193	<0.0001
HCV	Negative	188	1	189				
	Positive	4	455	459				
	Total	192	456	648	99.23	0.9814	0.9652 to 0.9976	<0.0001

Abbreviations: CAP/CTM, Cobas® AmpliPrep/Cobas® TaqMan®; HBV, hepatitis B virus; HCV, hepatitis C virus; CI, confidence interval.

The overall concordance rate between the results from the HepaTrio and the CAP/CTM assays for the detection of HBV along with HCV was 94.14%.

**Table 2:** Summary of discrepant cases between the HepaTrio and the CAP/CTM assays.

		HBV		HCV			Serologic tests^a^					PCR^b^		Final diagnosis		
Discrepancy in	N (%) of cases	CAP/CTM	HepaTrio	CAP/CTM	HepaTrio	HepaTrio HAV	HBsAg	Anti-HBc	Anti-HBs	Anti-HCV	Anti-HAV IgM	HBV	HCV	HBV	HCV	HAV
HBV	2 (0.31)	**Pos**	**Neg**	Neg	Neg	Neg	**Pos**	**Pos**	**Neg**	Neg	Neg or NT	**Pos**	NT	**Pos**	Neg	Neg
	1 (0.15)	**Pos**	**Neg**	Pos	Pos	Neg	Neg	**Pos**	Neg	Pos	NT	**Pos**	Pos	**Pos**	Pos	Neg
	9 (1.39)	**Pos**	**Neg**	Neg	Neg	Pos	**Pos**	**Pos**	**Neg**	Neg	Pos	**Pos**	NT	**Pos**	Neg	Pos
	3 (0.46)	**Neg**	**Pos**	Pos	Pos	Neg	Neg	**Pos**	Neg	Pos	Neg or NT	**Pos**	Pos	**Pos**	Pos	Neg
	1 (0.15)	**Pos**	**Neg**	Neg	Neg	Pos	**Neg**	Pos	**Pos**	Neg	Pos	**Neg**	NT	**Neg**	Neg	Pos
	2 (0.31)	**Pos**	**Neg**	Pos	Pos	Neg	Neg	Pos	**Pos**	Pos	Neg or NT	**Neg**	Pos	**Neg**	Pos	Neg
	7 (1.08)	**Neg**	**Pos**	Pos	Pos	Neg	Neg	Pos or NT	**Pos**	Pos	Neg or NT	**Neg**	Pos	**Neg**	Pos	Neg
	1 (0.15)	**Pos**	**Neg**	Pos	Pos	Neg	NT	NT	NT	Pos	NT	NT	Pos	Ind	Pos	Neg
	6 (0.93)	**Neg**	**Pos**	Pos	Pos	Neg	Neg or NT	NT	Pos or NT	Pos	Neg or NT	NT	Pos	Ind	Pos	Neg
HCV	2 (0.31)	Neg	Neg	**Pos**	**Neg**	Neg	Neg	Neg or Pos	Pos	**Pos**	NT	NT	**Pos**	Neg	**Pos**	Neg
	1 (0.15)	Neg	Neg	**Pos**	**Neg**	Pos	NT	NT	NT	**Pos**	NT	NT	**Pos**	Neg	**Pos**	Pos
	1 (0.15)	Pos	Pos	**Neg**	**Pos**	Neg	Pos	Pos	Neg	NT	NT	Pos	NT	Pos	Ind	Neg
HBV and HCV	1 (0.15)	**Neg**	**Pos**	**Pos**	**Neg**	Neg	**Neg**	**Neg**	**Pos**	**Pos**	Neg	**Neg**	**Pos**	**Neg**	**Pos**	Neg

Abbreviations: CAP/CTM, Cobas® AmpliPrep/Cobas® TaqMan®; HBV, hepatitis B virus; HCV, hepatitis C virus; HAV, hepatitis A virus; PCR, polymerase chain reaction; Pos, positive; Neg, negative; NT, not tested; Ind, indeterminate.

a Serologic test results between one month before and after sample collection were referenced when the results were consistent at two or more separated time points.

b Follow-up CAP/CTM HBV or HCV tests within a month.

**Figure 2:**
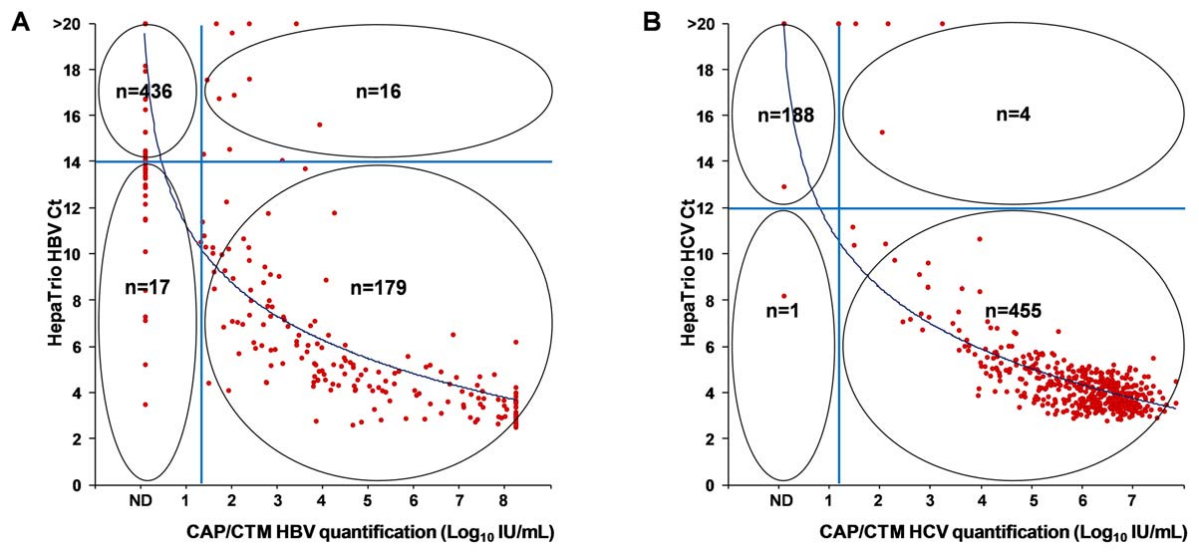
Correlation between cycle threshold (Ct) values on the HepaTrio test and viral concentrations. A. Serum levels of HBV DNA, which were quantified by the Cobas® AmpliPrep/Cobas® TaqMan® (CAP/CTM) HBV assay, correlated well with Ct values on the HepaTrio test (n = 648, *r* = −0.9230, 95% confidence interval [CI] = −0.9336 to −0.9107, *P*<0.0001). B. The correlation coefficient between Ct values on the HepaTrio test and serum HCV RNA concentrations measured by the CAP/CTM HCV assay was −0.8458 (n = 648, 95% CI = −0.8663 to −0.8223, *P*<0.0001). Filled red circles represent individual values and straight blue lines denote cutoff Ct values of the HepaTrio or lower detection limits of the CAP/CTM assays. ND, not detected.

Figure 2 illustrates the correlation between the Ct from the HepaTrio test and viral load quantified by the CAP/CTM assays. The Ct from the HepaTrio test was inversely correlated with the serum level of HBV DNA (*r* = −0.9230) and HCV RNA (*r* = −0.8458).

### 3. Clinical Performance of the Tests

According to the above-mentioned diagnostic accuracy criteria, all enrolled subjects were classified as true or false HBV and HCV infections, or indeterminate cases (Figure 1). The sensitivity and specificity of the HepaTrio test were 93.8% and 98.2%, respectively, for detecting HBV infections, after excluding the indeterminate cases (Table 3), and were 99.1% and 100.00%, respectively, for detecting HCV infection. The CAP/CTM HBV test showed a sensitivity of 98.5% and a specificity of 99.3% for detecting HBV infection in the same samples. The CAP/CTM HCV assay also demonstrated 100.0% sensitivity and specificity for the diagnosis of HCV infections (Table 3).

**Table 3:** Sensitivities and specificities of the HepaTrio test and the CAP/CTM assays for detecting HBV and HCV infections.

Target virus	Assay	Sensitivity (%)	95% CI (%)	Specificity (%)	95% CI (%)
HBV	CAP/CTM HBV^a^	98.45	95.55 to 99.68	99.33	98.05 to 99.86
	CAP/CTM HBV^b^	98.46	95.57 to 99.68	99.34	98.08 to 99.86
	HepaTrio^a^	93.81	89.44 to 96.76	98.21	96.50 to 99.22
	HepaTrio^b^	94.00	89.75 to 96.86	98.21	96.51 to 99.23
HCV	CAP/CTM HCV^a^	100.00	99.20 to 100.00	100.00	98.06 to 100.00
	CAP/CTM HCV^b^	100.00	99.20 to 100.00	100.00	98.07 to 100.00
	HepaTrio^a^	99.13	97.78 to 99.76	100.00	98.06 to 100.00
	HepaTrio^b^	99.13	97.79 to 99.76	100.00	98.06 to 100.00

Abbreviations: CAP/CTM, Cobas® AmpliPrep/Cobas® TaqMan®; HBV, hepatitis B virus; HCV, hepatitis C virus; CI, confidence interval.

a Sensitivity and specificity were calculated after excluding the cases with indeterminate final diagnosis (See Table 2).

b The diagnostic performance was estimated on the assumption that the evaluated assay yielded correct results for the indeterminate cases.

### 4. Detection of HBV and HCV Coinfection

The cases in which HBV DNA and HCV RNA were concurrently detected by either or both the HepaTrio and the CAP/CTM assays are summarized in Table 4. The HepaTrio test identified 21 such cases (3.2% of all 648 specimens), and six (0.9%) of them were true coinfections (median Ct for HBV DNA, 9.74; median Ct for HCV RNA, 5.65), and the other seven were false positives for HBV DNA with a median Ct of 13.29 (median Ct for HCV RNA, 3.59). HBV and HCV coinfection could not be identified by reviewing the medical records of the remaining eight patients (median Ct for HBV DNA, 12.54; median Ct for HCV RNA, 4.62) (Table 4).

**Table 4:** Summary of HBV and HCV coinfection cases detected by the HepaTrio and/or the CAP/CTM assays.

		CAP/CTM		HepaTrio			Serologic tests^a^					PCR^b^		Final diagnosis		
Coinfectiondetected by	N (%) of cases	HBV	HCV	HBV	HCV	HepaTrio HAV	HBsAg	Anti-HBc	Anti-HBs	Anti-HCV	Anti-HAV IgM	HBV	HCV	HBV	HCV	Coinfection
HepaTrio alone	3 (0.46)	Neg	Pos	**Pos**	**Pos**	Neg	Neg	**Pos**	**Neg**	**Pos**	Neg or NT	**Pos**	**Pos**	Pos	Pos	**Yes**
	7 (1.08)	Neg	Pos	**Pos**	**Pos**	Neg	Neg	Pos or NT	**Pos**	**Pos**	Neg or NT	**Neg**	Pos	Neg	Pos	No
	1 (0.15)	Pos	Neg	**Pos**	**Pos**	Neg	Pos	Pos	Neg	NT	NT	Pos	NT	Pos	Ind	Ind
	6 (0.93)	Neg	Pos	**Pos**	**Pos**	Neg	Neg or NT	NT	Pos or NT	Pos	Neg or NT	NT	Pos	Ind	Pos	Ind
CAP/CTM alone	1 (0.15)	**Pos**	**Pos**	Neg	Pos	Neg	Neg	**Pos**	**Neg**	Pos	NT	**Pos**	**Pos**	Pos	Pos	**Yes**
	2 (0.31)	**Pos**	**Pos**	Neg	Pos	Neg	Neg	Pos	**Pos**	Pos	Neg or NT	**Neg**	Pos	Neg	Pos	No
	1 (0.15)	**Pos**	**Pos**	Neg	Pos	Neg	NT	NT	NT	Pos	NT	NT	Pos	Ind	Pos	Ind
Both	3 (0.46)	**Pos**	**Pos**	**Pos**	**Pos**	Neg	**Pos**	**Pos**	**Neg**	Pos	NT	**Pos**	**Pos**	Pos	Pos	**Yes**
	1 (0.15)	**Pos**	**Pos**	**Pos**	**Pos**	Neg	Neg	NT	Neg	Pos	NT	NT	Pos	Ind	Pos	Ind

Abbreviations: HBV, hepatitis B virus; HCV, hepatitis C virus; CAP/CTM, Cobas® AmpliPrep/Cobas® TaqMan®; HAV, hepatitis A virus; PCR, polymerase chain reaction; Pos, positive; Neg, negative; NT, not tested; Ind, indeterminate.

a Serologic test results between one month before and after sample collection were referenced when the results were consistent at two or more separated time points.

b Follow-up CAP/CTM HBV or HCV tests within a month.

The CAP/CTM HBV and HCV assays yielded eight (1.2% among all 648 cases) results in which HBV DNA and HCV RNA were concurrently detected. Among them, four were true coinfections (median HBV DNA, 1.52×10^3^ IU/mL; median HCV RNA, 5.82×10^6^ IU/mL) and the other two were false positives for HBV DNA with results of 225 and 507 IU/mL (HCV RNA, 1.53×10^6^ and 1.19×10^4^ IU/mL, respectively). HBV and HCV coinfection could not be determined for the remaining two patients (HBV DNA of 22.0 and 77.8 IU/mL, HCV RNA of 5.76×10^4^ and 1.17×10^7^ IU/mL) (Table 4).

In addition, 7 (26.9%) out of 25 cases in which both HBV DNA and HCV RNA were detected by the HepaTrio test and/or by the CAP/CTM assays were true HBV and HCV coinfection, and both the HepaTrio and CAP/CTM tests concurrently indicated coinfection in only three of the seven cases.

### 5. Detection of HAV by the HepaTrio Test

The HepaTrio test detected 18 (2.8% among all 648 cases) positives for HAV RNA (Figure 1), and all of those cases were anti-HAV IgM-positive, except one in which HAV IgM was not assessed. Most of the HAV RNA-positive cases (16 of 18, 88.9%) were acute HAV infection in chronic HBV carriers. The median Ct for HAV RNA in those 18 cases was 4.59, and signals from the amplification of HAV RNA were not detected in any of the remaining 630 samples (Ct>20.0).

### 6. Validation of the Clinical Performance of the HepaTrio Test

A total of 100 samples from hemodialysis patients, which consisted of 53 males and 47 females, were assayed by the HepaTrio test. Median age of the patients was 58.5 years (first to third quartiles, 51.0 to 69.0 years) and median hemodialysis period was 51.5 months (first to third quartiles, 30.8 to >66.0 months).

For this patient group, the clinical performance of the HepaTrio test is summarized in Table 5. The prevalence of HBV and HCV infections was 3.0% and 6.0%, respectively, and there were no HBV and HCV coinfections or HAV infection cases. The median Ct on the HepaTrio test for the three true HBV and six true HCV infection cases was 4.85 and 2.48, respectively. There were seven false positive cases for HBV infection with a median Ct of 9.60, but no false negative cases were found. In addition, there was one false negative case for HCV infection (Ct>20.0), but no false positives.

**Table 5:** Diagnostic performance of the HepaTrio test for detecting HBV and HCV infections from a total of 100 hemodialysis patients.

Parameter	HBV	HCV
Prevalence (%)	3.0	6.0
Sensitivity (%)^a^	100.0 (29.2 to 100.0)	83.3 (35.9 to 99.6)
Specificity (%)^a^	92.8 (85.7 to 97.0)	100.0 (96.2 to 100.0)
PPV (%)^a^	30.0 (6.7 to 65.2)	100.0 (47.8 to 100.0)
NPV (%)^a^	100.0 (96.0 to 100.0)	98.9 (94.3 to 100.0)
Correct classification (%)^a^	93.0 (86.1 to 97.1)	99.0 (94.6 to 100.0)
Misclassification (%)^a^	7.0 (2.9 to 13.9)	1.0 (0.0 to 5.4)
Positive LR	13.86	∞
Negative LR	0.00	0.17

Abbreviations: HBV, hepatitis B virus; HCV, hepatitis C virus; PPV, positive predictive value; NPV, negative predictive value; LR, likelihood ratio.

a Data are shown as % (95% confidence interval).

## Discussion

In this study, we evaluated the clinical performance of a novel multiplex real-time PCR assay, which was designed to simultaneously detect HAV, HBV and HCV. Although there have been some studies on multiplex assays for the concurrent detection of HBV, HCV, and human immunodeficiency virus, which are major targets in screening tests for donated blood [20]–[25], there has been no real-time PCR assay for the concurrent detection of the three common hepatitis viruses. In a study, a flow-cytometric microsphere-based hybridization assay after reverse-transcription PCR was developed, and the assay provided a sensitive and specific concurrent detection of HIV, HBV, and HCV from 35 serologically well-established clinical specimens as well as from 20 seronegative blood donor plasma samples [20]. Another study reported that an automated multiplex real-time PCR assay system demonstrated 0.34% of false-positive rate in the concurrent detection of HIV, HBV, and HCV from 55,537 pooled blood donor plasma samples [21]. A qualitative visual DNA chip method combined with multiplex and nested PCR was also developed for the detection of the same three viruses and this assay showed an analytical sensitivity of around 1 pg of viral DNA [22]. More recently, another assay utilizing transcription-mediated amplification (TMA) and chemiluminescent detection principles demonstrated a specificity of greater than 99% in the detection of the three viruses from 740 seronegative samples [25]. Although these assays utilized different methods, reports on the assays mainly focused on the evaluation of the specificity and analytical sensitivity, since they were developed for screening donated blood. Meanwhile, our study’s main focus was on the evaluation on the clinical sensitivity and specificity of the new multiplex real-time PCR assay using clinical samples.

Since the efficacies of methods for amplifying DNA and RNA from different viruses would be hard to equalize, developing a multiplex PCR assay could be very challenging. The HepaTrio test is novel in that it is performed by multiplex reverse transcription real-time PCR to detect the three common hepatitis viruses. This assay utilizes two distinctive features, the Real Amplicon Detection (READ) and the previously mentioned DPO technology. The READ is a type of real-time PCR which utilizes a different technique from conventional fluorescence detection methods used in other real-time PCR assays. The manufacture of the HepaTrio suggests that the READ technology enables early detection of amplicons, thus the Ct from the HepaTrio tests in our data was generally lower than that from a conventional real-time PCR. However, detailed principle of the READ is undisclosed by the manufacturer.

Meanwhile, the HepaTrio demonstrated well-agreed results with those by widely used CAP/CTM assays, especially for the detection of HCV RNA. This test also showed sensitivity and specificity greater than 99.0% for detecting HCV infection from patients suspected of having acute hepatitis, and the test yielded positive results for HAV RNA in anti-HAV IgM-positive samples. The sensitivity of the HepaTrio test for HBV infection from the same subjects was 93.8%, and thus improvement is required. Interestingly, the Ct with the HepaTrio test correlated well with the viral load. Therefore, the Ct of this assay may be used as a proxy for viral load when quantitative PCR tests are not available.

Together with the ability to detect three hepatitis viruses concurrently, the HepaTrio test is advantageous in that the minimum amount of sample required per test is as small as 500 µL. The minimum sample volumes required for the CAP/CTM HBV test v2.0 and the CAP/CTM HCV test, respectively, are 500 µL and 850 µL, excluding the dead volume. Hence, about three times more quantity of sample is needed for the CAP/CTM assays than for the HepaTrio test when testing for both HBV and HCV. However, the CAP/CTM tests still have the advantage of quantitative determination of viral nucleic acids.

In our results for HBV, the median Ct of the eight (1.2% among all cases) HepaTrio false-positive cases was 13.15 (first to third quartiles, 12.55 to 13.45; cutoff, 14.0), and all of these cases were HCV infection with a median HCV RNA level of 1.06×10^5^ IU/mL. Thus, a Ct for HBV DNA around the cutoff could be false-positives, and these may be due to interference in the detection of signals from different fluorophores or from cross-reaction during the amplification of nucleic acids. Both possible causes could be an inherent limitation of a multiplex assay, and need to be resolved. Although we could not obtain more detailed information on the above-mentioned READ technology because the manufacturer has not made this information public, it is also a real-time PCR principle and we expect that this technology can be technically improved more by further researches.

The median Ct from the HepaTrio test for the 12 (1.9% among all) HBV false-negative cases was 17.23 (first to third quartiles, 15.35 to 19.70), and the median HBV DNA level in these cases was 107 IU/mL (first to third quartiles, 50.6 to 714 IU/mL). There were also four (0.6% among all) HCV false-negative cases with the HepaTrio test. The median HCV RNA concentration in those four cases was 126 IU/mL. Therefore, the false-negative results on the HepaTrio test could be due to the lower analytical sensitivity of this assay compared to that of the CAP/CTM assays. The lower limits of detection (LOD) suggested by the manufacturer are 20 IU/mL for HBV and 30 IU/mL for HCV, which are similar or a little higher compared to those of the CAP/CTM assays. Considering the low virus levels in those false-negative cases, the HepaTrio was able to detect most of cases with high or usual viral loads, thus this assay may have good sensitivity when assaying samples from typical hepatitis patients. However, specimens with various concentrations of HBV or HCV can be encountered in a clinical laboratory, thus the HepaTrio test would be more useful when the analytical sensitivity for detecting HBV DNA is improved.

In this study, a total of seven HBV and HCV coinfection cases were found by both or either the HepaTrio test and the CAP/CTM assays, and thus the HBV and HCV coinfection rate in our population was 1.1%, which is lower than that reported by some previous studies [9]–[11]. These differences are likely the result of differences in the populations and in the methods utilized for detecting coinfection between the studies. In our seven coinfection cases, the median serum level of HBV DNA was 5.28×10^2^ IU/mL and that of HCV RNA was 4.67×10^4^ IU/mL. Therefore, most HBV and HCV coinfection cases seem to be caused by acute HCV superinfection in chronic HBV carriers since Korea has a high prevalence of chronic hepatitis B. In contrast to our results, a previous study reported 40 cases of fulminant/subfulminant hepatitis, five (12.5%) of which were acute coinfection with HBV and HCV, and three (7.5%) had HCV superinfection [26]. Another study also reported a higher acute coinfection rate (9.4%) than HCV superinfection rate (3.1%) of chronic hepatitis B among 25 patients with fulminant/subfulminant hepatitis [27]. True diagnoses for nine cases in the present study, in which both HBV and HCV were positive by the HepaTrio test and/or the CAP/CTM assays, could not be identified because of insufficient evidence. Thus, the HBV and HCV coinfection rate in our population might actually be higher than indicated by our results.

The HepaTrio test showed high sensitivity and NPV for detecting HBV infection as well as high specificity and PPV for the diagnosis of HCV infection in an independent high-risk population. However, PPV for HBV infection was low likely due to the low prevalence (3.0%) of HBV infection in the evaluated population and relatively small size of the population as well as the false-positive results. Although PPV is not intrinsic to the test and depends on the prevalence of a target disease, the current version of the HepaTrio test would need to be improved - especially for the detection of HBV when the test is intended to be used as a tool for screening hepatitis. A recent study on 353 hemodialysis patients also reported similar prevalence of HBV or HCV infection (4.5% and 8.5%, respectively) [28]. Further large-scale studies with high-risk subjects are needed to confirm the usefulness of the HepaTrio test in the population screening setting. In addition, considering diagnostic accuracy as well as cost-effectiveness, studies on the advantages of the multiplex PCR assay over the widely used serologic tests for the diagnosis of hepatitis would also be necessary.

Results from an assay detecting viruses could be affected by the genotypes of the viruses. Although the genotypes of the detected HBV were not further identified, the most prevalent HBV genotype in Korea was C2, which accounted for 98.1% of the total HBV cases in a recent domestic study [29]. Similar to our data on the HCV genotypes, genotypes 1b and 2a were the two most common types of HCV in a previous study, accounting for virtually equal proportions, and other genotypes were extremely rare [30]. Therefore, our data could be regarded as obtained from the HBV genotype C and HCV genotypes 1 and 2. Further evaluation with genotype-determined cases may be helpful for assessing the clinical performance of the HepaTrio test according to the HBV and HCV genotypes.

In our pilot study, the HepaTrio qualitative real-time PCR test demonstrated good clinical performance for the concurrent detection of the three hepatitis viruses, and results from the HepaTrio test agreed well with those of the widely used CAP/CTM quantitative HBV and HCV assays, although this new assay would require improvement in the detection of HBV infections. This test would be used as an ancillary tool for the diagnosis of hepatitis when a patient is suspected to having hepatitis rather than as a screening test. As a multiplex assay, the usefulness of this new test also would need to be further evaluated by comparing its cost-effectiveness and diagnostic performance with those of serologic tests in the concurrent screening of acute hepatitis A, B and C.
